# Dietary Resveratrol Prevents the Development of Food Allergy in Mice

**DOI:** 10.1371/journal.pone.0044338

**Published:** 2012-09-04

**Authors:** Yui Okada, Kyoko Oh-oka, Yuki Nakamura, Kayoko Ishimaru, Shuji Matsuoka, Ko Okumura, Hideoki Ogawa, Masashi Hisamoto, Tohru Okuda, Atsuhito Nakao

**Affiliations:** 1 Department of Immunology, University of Yamanashi Faculty of Medicine Chuo, Yamanashi, Japan; 2 The Institute of Enology and Viticulture, University of Yamanashi, Koufu, Yamanashi, Japan; 3 Deparment of Pathology, Juntendo University School of Medicine, Bunkyo-ku, Tokyo, Japan; 4 Atopy Research Center, Juntendo University School of Medicine, Bunkyo-ku, Tokyo, Japan; Charité-University Medicine Berlin, Germany

## Abstract

**Background:**

Resveratrol is a bioactive polyphenol enriched in red wine that exhibits many beneficial health effects via multiple mechanisms. However, it is unclear whether resveratrol is beneficial for the prevention of food allergy. This study investigated whether resveratrol inhibited the development of food allergy by using a mouse model of the disease.

**Methodology/Principal Findings:**

Mice fed standard diet or standard diet plus resveratrol were sensitized by intragastric administration of ovalbumin (OVA) and mucosal adjuvant cholera toxin (CT). Several manifestations of food allergy were then compared between the mice. The effects of resveratrol on T cells or dendritic cells were also examined by using splenocytes from OVA-specific T cell-receptor (TCR) transgenic DO11.10 mice or mouse bone marrow-derived dendritic cells (BMDCs) *in vitro*. We found that mice fed resveratrol showed reduced OVA-specific serum IgE production, anaphylactic reaction, and OVA-induced IL-13 and IFN-ã production from the mesenteric lymph nodes (MLNs) and spleens in comparison to the control mice, following oral sensitization with OVA plus CT. In addition, resveratrol inhibited OVA plus CT-induced IL-4, IL-13, and IFN-ã production in splenocytes from DO11.10 mice associated with inhibition of GATA-3 and T-bet expression. Furthermore, resveratrol suppressed the OVA plus CT-induced CD25 expression and IL-2 production in DO11.10 mice-splenocytes in association with decreases in CD80 and CD86 expression levels. Finally, resveratrol suppressed CT-induced cAMP elevation in association with decreases in CD80 and CD86 expression levels in BMDCs.

**Conclusions/Significance:**

Ingestion of resveratrol prevented the development of a food allergy model in mice. Given the *in vitro* findings, resveratrol might do so by inhibiting DC maturation and subsequent early T cell activation and differentiation via downregulation of CT-induced cAMP activation in mice. These results suggest that resveratrol may have potential for prophylaxis against food allergy.

## Introduction

Food allergy is defined as an immune-mediated pathological reaction toward food antigens, which is a major health concern and epidemic in developed countries [1]–[3]. Generally, the mucosal immune system in the intestine is hyporesponsive to innocuous food antigens (termed “oral tolerance”) due to different T cell events, such as anergy, clonal deletion, and the induction of regulatory T cells [4]. In contrast, mucosal sensitization to a food antigen occurs in patients with food allergy (a breakdown of the natural oral tolerance), which results in the antigen (allergen)-specific IgE production although the mechanisms are poorly understood [5], [6]. The development of new approaches to prevent the disease is of significant importance for public health because allergen avoidance is currently the only way available for prevention of food allergy [7].

Resveratrol (*trans*-3, 4¢, 5, -trihydroxystilbene) is a polyphenolic compound that is abundant in grapes and red wine and functions as a phytoalexin that protects against fungus infections in plants [8], [9]. Resveratrol has a wide range of biological and pharmacological activities in animal disease models and human health and diseases, including anti-inflammatory, anti-oxidative, and anti-carcinogenic properties via multiple molecular mechanisms [10]. Recently, resveratrol is of great interest since this reagent is reported to improve health and survival of mice on a high-calorie diet without apparent toxicity [11]. However, in spite of these extensive studies on the roles of resveratrol in human health and diseases, it remains unclear whether resveratrol is also beneficial for food allergy.

The primary aim of this study was to investigate whether ingestion of resveratrol inhibits mucosal sensitization to a food antigen, thereby suppressing the hallmark manifestations of food allergy such as antigen-specific IgE production, anaphylactic reaction, and Th2 cytokine production. For this purpose, we examined the effects of dietary resveratrol on a mouse model of food allergy induced by oral administration of ovalbumin (OVA) with mucosal adjuvant cholera toxin (CT) [12].

## Results

### Resveratrol Inhibits CT-driven Mucosal Sensitization to OVA in Mice

**Figure 1:**
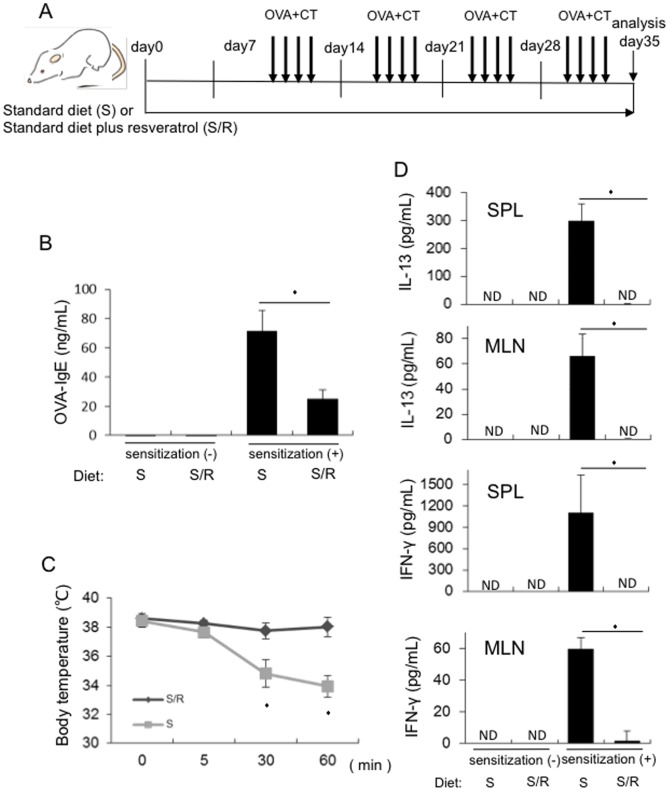
Resveratrol inhibits CT-driven mucosal sensitization to OVA in mice. Mice were fed the standard diet or standard diet plus resveratrol (22.4 mg/kg diet, 0.01% resveratrol) for 5 weeks (day 0–35). The mice were orally administered 50 mg of OVA with 10 µg of CT 1 week after the start of resveratrol feeding and 4 times a week for 4 weeks and sacrificed at the 5 weeks (on day 35) for the analysis below. A. Experimental protocol. B. Serum samples were collected on day 35 from the standard diet-fed and standard diet plus resveratrol-fed mice with or without OVA plus CT sensitization. OVA-specific serum IgE concentrations were measured by ELISA (n = 7 per group). C. The standard diet-fed and standard diet plus resveratrol-fed mice sensitized with OVA plus CT were challenged intraperitoneally with OVA on day 35 and rectal temperatures were measured with a digital thermometer at 0, 5, 30, and 60 minutes after the challenge (n = 10 per group). D. Splenocytes (SPL) and mesenteric lymph node (MLN) cells were obtained on day 35 from the standard diet-fed and standard diet plus resveratrol-fed mice with or without OVA plus CT sensitization and the cells were re-stimulated *in vitro* with OVA for 3 days (n = 5 per group). The culture supernatants were then collected and IL-13 and IFN-ã concentrations were measured by ELISA. (S: standard diet-fed mice, S/R: standard diet plus resveratrol-fed mice) Values represent the mean ± SD. *P<0.05 in comparison to the corresponding controls. Representative results from 2 independent experiments with same results are shown.

The mice were fed the standard diet or standard diet plus resveratrol (22.4 mg/kg diet, 0.01% resveratrol) for 5 weeks. The mice were orally administered 50 mg of OVA with 10 µg of CT 4 times a week for 4 weeks, beginning 1 week after the start of resveratrol administration (**Figure 1A**) and then were sacrificed at 5 weeks for the analysis as was noted below. The feeding dose was determined based on a study showing improved longevity of mice by chronic dietary resveratrol ingestion without toxicity [11].

Mice fed the standard diet showed the induction of serum OVA-specific IgE production following oral sensitization with OVA plus CT (**Figure 1B**) as previously described [12]. In contrast, the mice fed a standard diet plus resveratrol showed a decrease in OVA-specific IgE production after oral sensitization (**Figure 1B**). Consistent with the findings of the serum IgE levels, resveratrol-fed mice sensitized with OVA plus CT showed a smaller drop in the extent of rectal temperatures than the control mice sensitized with OVA plus CT, upon intraperitoneal OVA challenge (**Figure 1C**). In addition, OVA-specific IL-13 and IFN-ã productions from splenocytes and mesenteric lymph nodes (MLN)-derived cells were decreased in resveratrol-fed mice in comparison to those in control mice, following sensitization with OVA plus CT (**Figure 1D**). The frequencies of apoptotic cells in splenocytes and MLN cells freshly isolated from the mice fed standard diet and the mice fed the standard diet plus resveratrol were comparable based on propidium iodide (PI) and Annexin V staining **(Figure S1 A and B**). In addition, the frequencies of apoptotic cells in splenocytes and MLN cells from the mice fed standard diet and the mice fed the standard diet plus resveratrol were comparable following *in vitro* OVA re-stimulation based on PI and Annexin V staining (**Figure S1 C**). These data suggested that dietary resveratrol did not affect the cellular viability in spleen and MLN cells in mice. There were no significant changes in body weights and food or water intake in resveratrol-fed mice in comparison to those in control mice (data not shown). These results suggest that resveratrol inhibited CT-driven mucosal sensitization to OVA in mice without apparent toxicity in association with inhibition of OVA-specific both Th1 and Th2 differentiation.

### Resveratrol Inhibits OVA Plus CT-induced Th1 and Th2 Differentiation *in vitro*

The effects of resveratrol on OVA plus CT-stimulated spleen cells from OVA-specific TCR transgenic DO11.10 mice were examined to investigate how resveratrol inhibited CT-driven mucosal sensitization to OVA *in vivo* [13], hoping to mimic the effects of resveratrol *in vitro*. It is not possible to exactly measure the concentrations of resveratrol in the target tissues in mice (e.g. gut-associated lymphoid tissue: GALT); therefore, this study used the concentrations of resveratrol that can be attained by red wine intake (∼10 µM) in humans [9] for the *in vitro* studies.

**Figure 2:**
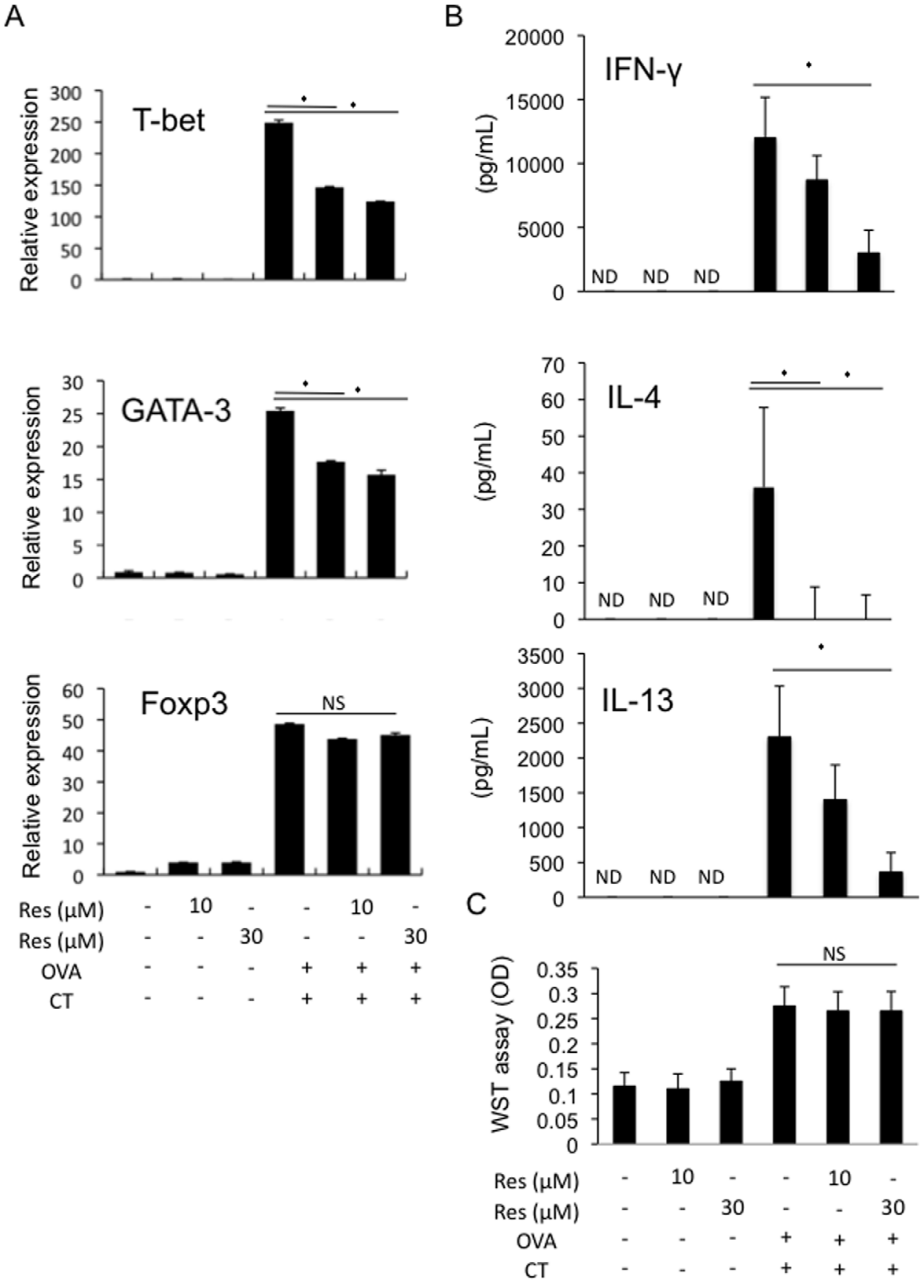
Resveratrol inhibits OVA plus CT-induced Th1/2 cell differentiation *in vitro*. DO11.10 mice-derived splenocytes were un-stimulated or stimulated with 300 µg/ml OVA plus 12 pM CT and were cultured in the presence or absence of 10 or 30 µM resveratrol for the indicated times. Then the analysis described below was performed. A. After 3 day-culture, RNA samples were extracted from the cells and the cDNA samples were synthesized using reverse transcriptase system. Quantitative real-time PCR analysis was then done for T-bet, GATA3, and Foxp3 mRNAs. Relative expression levels are shown (n = 3 per group). B. After 3 day-culture, the supernatants were collected and IFN-ã, IL-4, and IL-13 concentrations were measured by ELISA (n = 9 per group). C. After 3 day-culture, the cells were subjected to a WST assay for evaluation of the cell viability (n = 4 per group). Values represent the mean ± SD. *P<0.05 in comparison to the corresponding controls. (ND: non-detected).

DO11.10 mice-splenocytes stimulated with OVA plus CT showed increased IFN-ã, IL-4, and IL-13 production in association with robust increases in mRNA levels of T-bet and GATA3, master transcriptional regulators of Th1 and Th2 differentiation, respectively (**Figure 2A and B**). The addition of 10 or 30 µM resveratrol inhibited those responses (**Figure 2A and B**). In contrast, we found that mRNA expression levels of Foxp3, a master regulator of regulatory T cells (Tregs) were not affected by the resveratrol treatment (**Figure 2A**). In addition, the treatment of DO11.10 mice-splenocytes with 10 or 30 µM resveratrol did not affect the cell viability based on trypan blue dye exclusion (**Figure S2**) and WST assay (**Figure 2C**), suggesting that the inhibition of Th1/Th2 differentiation by resveratrol was not due to a reduced cell viability by resveratrol. These results indicated that resveratrol inhibited OVA plus CT-induced both Th1 and Th2 differentiation *in vitro* as well as *in vivo*.

### Resveratrol Inhibits Early T Cell Activation Associated with Decrease of Co-stimulatory Molecule Expression in Antigen-presenting Cells (APCs)

The *in vitro* results suggest that resveratrol may inhibit OVA plus CT-induced T cell responses at the level of early T cell activation, thereby suppressing subsequent Th1 and Th2 cell differentiation. Therefore, the effects of resveratrol on early T cell activation markers, surface CD25 expression and IL-2 production, were examined by using DO11.10 mice-splenocytes *in vitro*.

**Figure 3:**
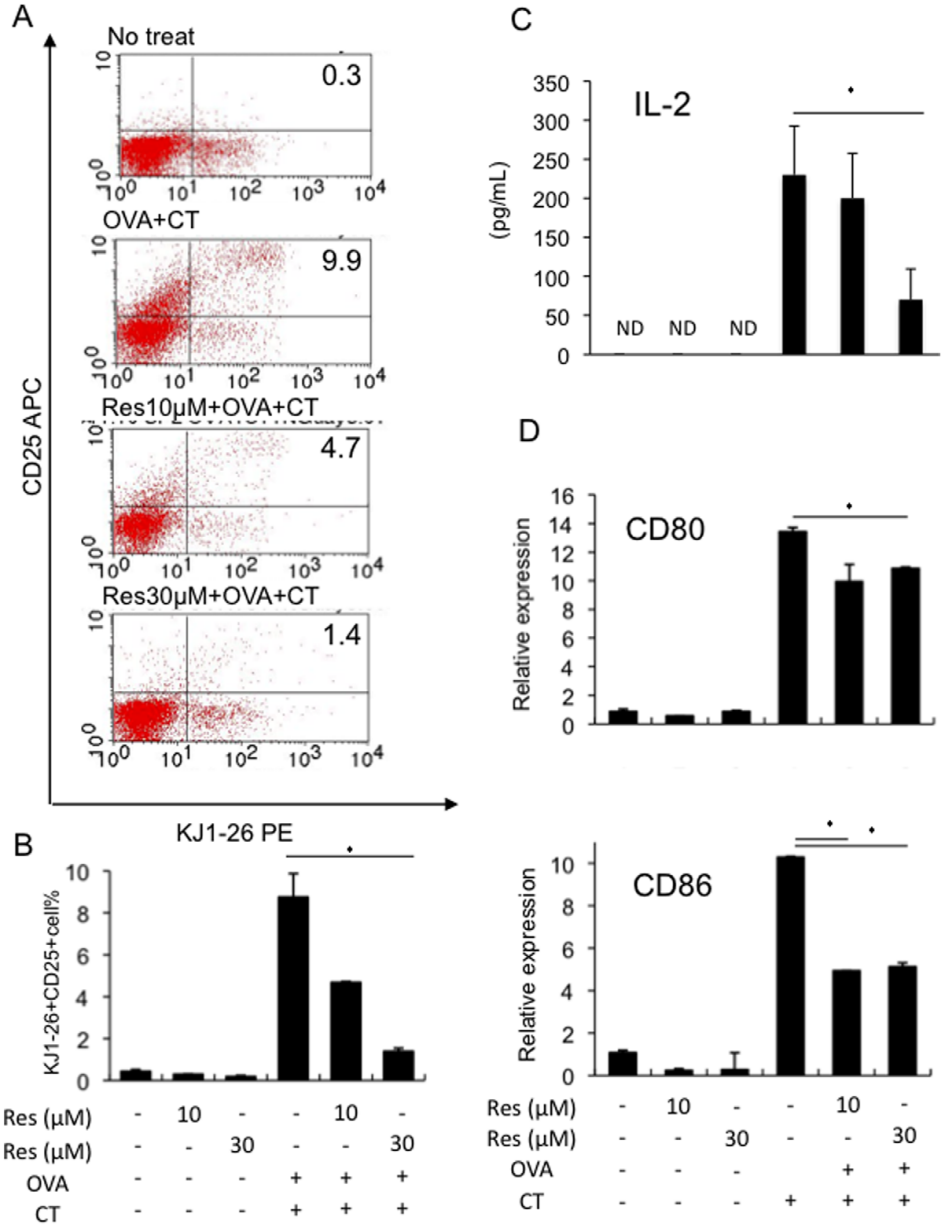
Resveratrol inhibits early T cell activation and co-stimulatory molecule expression in APCs *in vitro*. DO11.10 mice-derived splenocytes were un-stimulated (no treat) or stimulated with 300 µg/ml OVA plus 12 pM CT and were cultured in the presence or absence of 10 or 30 µM resveratrol for 1 day. Then the analysis described below was performed. A. After 1 day-culture, whole splenocytes were stained with PE-conjugated anti-KJ1–26 and APC-conjugated anti-CD25 antibody. Representative FACS plots are shown. Numbers indicate the proportion of the KJ1–26^+^ CD25^+^ cells. B. Quantitative analysis of A (n = 3 per group). C. After 1 day-culture, the supernatants were collected and IL-2 concentrations were measured by ELISA (n = 9 per group). D. After 1 day-culture, RNA samples were extracted from the splenocytes and the cDNA samples were synthesized using reverse transcriptase system. Quantitative real-time PCR analysis was then performed to assess CD80 and CD86 mRNA levels. Relative expression levels are shown (n = 3 per group). Values represent the mean ± SD. *P<0.05 in comparison to the corresponding controls. (ND: non-detected).

DO11.10 mice-derived splenocytes stimulated with OVA plus CT showed increased surface expression levels of CD25 in OVA-specific CD4^+^ T cells that were identified by staining with the anti-clonotypic antibody KJ1–26 (**Figure 3A and B**). Treatment of the cells with resveratrol (10 and 30 µM) decreased the frequency of CD25^+^ KJ1–26^+^ cells (**Figure 3A and B**). In addition, DO11.10 mice-derived splenocytes stimulated with OVA plus CT showed increased IL-2 production in the culture supernatants, which were inhibited by the addition of 30 µM resveratrol (**Figure 3C**). Furthermore, OVA plus CT-induced mRNA expression of co-stimulatory molecules CD80 and CD86 were decreased by resveratrol in DO11.10 mice-splenocytes (**Figure 3D**). These data suggest that resveratrol inhibited OVA plus CT-induced early T cell activation by decreasing the expression levels of co-stimulatory molecules in antigen presenting cells (APCs), most likely in DCs.

### Resveratrol Inhibits CT-induced cAMP Elevation in BMDCs

The adjuvant activity of CT is attributed, at least in part, to up-regulation of co-stimulatory molecules in APCs [14], [15]. It is thought that CT-induced intracellular cAMP elevation is involved in the up-regulation of co-stimulatory molecules in APCs and licenses them for Th2 priming [16]–[18]. Therefore, the effects of resveratrol on cAMP elevation induced by CT in bone marrow-derived dendritic cells (BMDCs) were examined.

**Figure 4:**
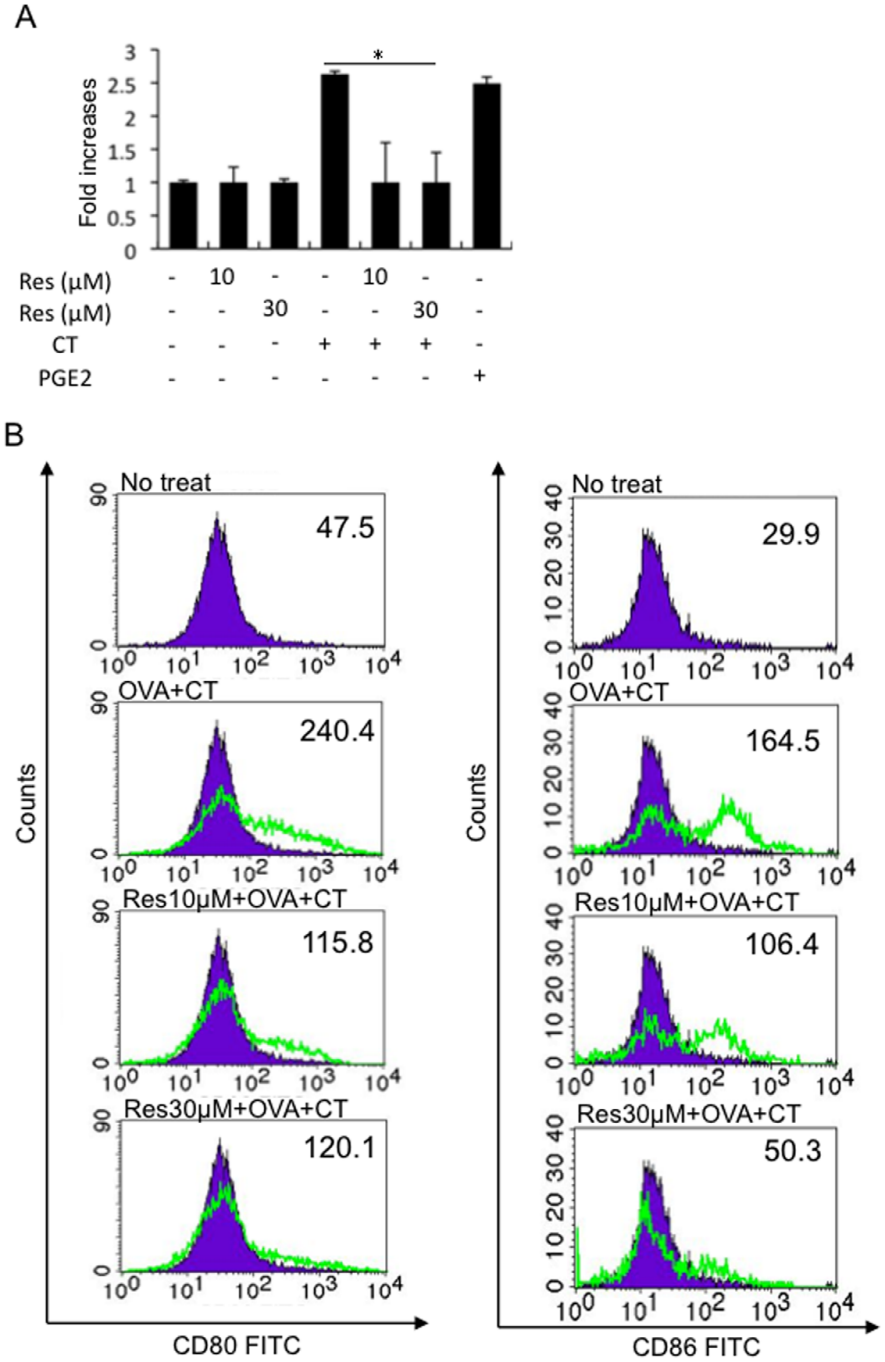
Resveratrol inhibits CT-induced cAMP elevation in BMDCs. A. Cultured BMDCs were un-stimulated or stimulated with 12 pM CT in the presence or absence of 10 or 30 µM resveratrol for 1 hour or 10 nM PGE2 (as a positive control) and intracellular cAMP levels were measured using a cAMP EIA kit. Relative changes in intracellular cAMP levels are indicated. Values represent the mean ± SD (n = 3 per group). B. Cultured BMDCs were un-stimulated (no treat) or stimulated with 300 µg/ml OVA plus 12 pM CT and were cultured in the presence or absence of 10 or 30 µM resveratrol for 3 days. The cells were then stained with APC-conjugated anti-CD11c and FITC-conjugated anti-CD80 or CD86 antibody and were subjected to FACS analysis. A representative histogram regarding surface CD80 and CD86 expression levels on CD11c gated cells is shown. Numbers indicate the mean fluorescence intensity (MFI) of CD80 and CD86-stained cells. The filled histogram indicates un-stimulated (no treat) BMDCs for comparison. Representative results from 3 independent experiments with similar results are shown.

CT or prostaglandin E2 (PGE2) (as a positive control) induced cAMP elevation in BMDCs (**Figure 4A**) as reported previously [16]. Resveratrol (10 and 30 µM) inhibited CT-induced cAMP elevation in BMDCs (**Figure 4A**). In addition, OVA plus CT-induced surface CD80 and CD86 expression levels in BMDCs were decreased by 10 and 30 µM resveratrol as indicated by mean fluorescence intensity (**Figure 4B**). The treatment of BMDCs with 10 or 30 µM resveratrol did not affect the cell viability based on a WST assay (data not shown). These results suggest that resveratrol inhibits CT-induced cAMP elevation in BMDCs in association with decreases in the expression levels of co-stimulatory molecules.

**Figure 5:**
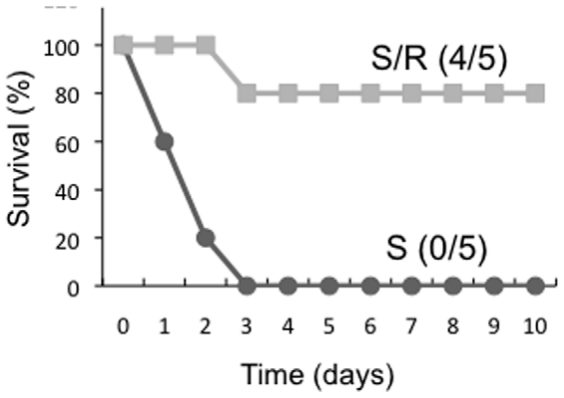
Dietary resveratrol protects mice against a lethal dose of CT. Survival of 8-week-old C57BL/6 mice orally challenged with CT at a dose of 150 µg monitored until day 10 after challenge (n = 5 per group). Representative results from 2 independent experiments with similar results are shown. (S: standard diet-fed mice, S/R: standard diet plus resveratrol-fed mice).

### Dietary Resveratrol Protects Mice Against a Lethal dose of CT

We thought that, if resveratrol inhibits the adjuvant activity (e.g. cAMP elevation) of a low-dose of CT as described above, resveratrol might also protect mice against the toxic effects of a high-dose of CT. Thus, we orally administered a high-dose of CT that causes severe toxicity to naïve mice fed with or without resveratrol.

One hundred percent of the mice fed the standard diet died by day 5 after a single challenge of 150 µg CT as previously described [19]. In contrast, 80% of the mice fed a standard diet plus resveratrol survived after the single challenge of CT (**Figure 5**).

These results suggest that dietary resveratrol protects mice against a lethal toxicity of CT at a high-dose, which may be related with the inhibitory effects of resveratrol on CT activity.

## Discussion

This study demonstrated that ingestion of resveratrol prevented CT-driven mucosal sensitization to OVA in mice without apparent toxic effects. The *in vitro* experiments showed that resveratrol was able to inhibit CT-induced cAMP elevation and maturation of DC and abrogated T cell activation and subsequent Th1/Th2 differentiation. The adjuvant activity of CT is largely attributed to its ability to induce co-stimulatory molecules in APCs via cAMP elevation [14], [15], [18]. Therefore, the current data suggest that resveratrol suppression of CT adjuvant activity is likely to be a key event associated with the inhibition of CT-induced mucosal sensitization to OVA *in vivo*. The findings that dietary resveratrol protected mice against a lethal dose of CT may support the hypothesis.

As stated above, the *in vivo* effects of resveratrol might be attributed to suppression of CT-induced DC maturation, which could occur in the gut-associated lymphoid tissue (GALT). However, the precise mechanisms by which resveratrol inhibits OVA-induced immune responses *in vivo* remain to be determined because resveratrol has a wide range of biological and pharmacological activities via multiple molecular mechanisms [10] and the bioavailability of resveratrol in mice (and also in humans) remains a major consideration [20]. In particular, further investigation should determine if it is possible to achieve the concentrations (10–30 µM) of resveratrol (that were used in the current *in vitro* studies) in the gut local environment in the resveratrol-fed mice.

Resveratrol treatment decreased OVA plus CT-induced co-stimulatory molecule expression levels in DO11.10 mice-splenocytes and in BMDCs. In consistent with this study, Sharma et al. previously reported that resveratrol at 20 µM suppressed the expression of co-stimulatory molecules (e.g. CD80) in LPS-stimulated mouse peritoneal macrophages *in vitro*, although the molecular mechanism was not addressed [21].

How resveratrol inhibits CT-induced cAMP elevation in BMDCs remains to be determined. Recently, Morinaga et al. reported that resveratrol inhibited CT-induced cAMP accumulation in Vero cells [22]. They suggested that resveratrol suppressed the CT activity indirectly by suppressing the internalization of CT or directly by interacting with CT and inhibiting its enzymatic activity to activate adenylate cyclase. These mechanisms might also occur in DC in this study.

Masilamani et al. and Zuercher et al. reported that dietary soybean isoflavons or polyphenol-enriched apple extract protected against CT-driven mucosal sensitization to peanut or OVA in mice, respectively [23], [24]. Masilamani et al. also showed that soybean isoflavons inhibited CT-induced DC maturation and subsequent T cell activation [23], which is similar to the current findings in case of resveratrol. Therefore, dietary polyphenols present in vegetables and fruits might have the general potential to prevent food allergy and other allergic diseases [20]. The current study identifies resveratrol as a new dietary polyphenol having such an anti-allergic activity and shows that CT adjuvant activity is likely to be an important target of resveratrol in the context of a food allergy model.

In summary, this study suggests that dietary resveratrol can prevent mucosal sensitization to food antigens without apparent toxic effects. Resveratrol has beneficial properties in animal disease models and human health and, as such, many clinical trials are on going with relative safety [10], [25]. The current results provide additional evidence that this remarkable natural substance may also be beneficial for prophylaxis against food allergy, where unmet preventive/therapeutic needs are greatest in allergic diseases.

## Methods

### Mice

Female 4–6 weeks Balb/c were purchased from Japan SLC (Tokyo, Japan) and DO11.10 transgenic mice [13] with Balb/c genetic backgrounds were bred under specific pathogen-free conditions. Susceptibility of mice to a lethal dose of CT was tested using 8 week-old C57BL/6 mice (Japan SLC, Tokyo, Japan). All animal experiments were approved by the Institutional Review Board of the University of Yamanashi.

### Dietary Resveratrol

The mice were maintained on a standard mouse diet for 2 weeks prior to the start of the experiment. All mice were housed 4–5 per cage with free access to food and water for 24 hours, under a 12∶12 light/dark cycle. Mice were fed the standard diet or resveratrol (>99%, Sigma-Aldrich, St Louis, MO)-mixed standard diet (22.4 mg/kg diet, 0.01% resveratrol) for 5 weeks as previously described [11]. The diet was provided in cages for no more than 1 week. Food intake and body weight were measured on a weekly basis for the duration of the study.

### Mucosal Sensitization

The mice were orally sensitized with ovalbumin (OVA) at 1 week after the start of resveratrol feeding as previously described [12] with some modifications. Briefly, mice were administered 50 mg of OVA (Sigma-Aldrich, St Louis, MO) with 10 µg of CT (List Biologicals, Campbell, CA) in a final volume of 200 µl by using a mouse feeding needle 4 times a week for 4 weeks.

### Serum IgE Levels

Serum levels of OVA-specific IgE levels were determined using the mouse OVA-specific IgE ELISA kit at week 5 (DS Pharma Biomedical, Ohsaka, Japan), according to the manufacturers’ instructions.

### Measurement of Anaphylaxis

All mice received a bolus i.p. challenge with OVA (100 µg) at week 5. Anaphylaxis was assessed in challenged mice by measuring changes in rectal temperature with a digital thermometer (Shibaura Electronics, Tokyo, Japan) at 0, 5, 30, 60 minutes after the challenge as previously described [26].

### Antigen-recall Response

The splenocytes and mesenteric lymph node (MLN) cells were obtained from the mice at week 5 and were cultured (2×10^6^ cells/well) for 72 hours in the presence of 300 µg/ml OVA. In the absence of OVA, IL-13 and IFN-ã production in the culture supernatants was not detected (data not shown).

### *In vitro* Cell Culture

Bone marrow-derived dendritic cells were generated from the femoral bone marrow cells of female mice as previously described [27]. DO11.10 mice derived-splenocytes or BMDCs were cultured in RPMI1640 (Sigma-Aldrich, St Louis, MO) containing 10% FCS and antibiotics. For experiments, the cells (1×10^6^ cells/well) were stimulated with 300 µg/ml OVA and 12 pM CT and were cultured for the indicated times. Resveratrol (10 and 30 µM) dissolved in DMSO or DMSO alone was added to the culture 30 minutes before the OVA plus CT stimulation. For some experiments, BMDCs were stimulated with 12 pM CT alone.

### Quantitative Real-time PCR

Total RNA was extracted from the cells using RNAeasy Mini kit (QUAGEN Inc., Valencia, CA). cDNA was then synthesized from 4 µg of total RNA using Reverse Transcriptase System (Applied Biosystems, Foster City, CA). Quantitative real-time PCR with specific primers and probes for mouse T-bet, GATA3, Foxp3, CD80, CD86, and GAPDH (Applied Biosystems, Foster City, CA) was then performed using the AB7300 real-time PCR system (Applied Biosystems) as previously described [28].

### ELISA

The amounts of IL-2, IL-4, IL-13, and IFN-ã in the culture supernatants were determined by using the mouse IL-2, IL-4, IL-13, and IFN-ã ELISA kits (eBioscience, San Diego, CA).

### Cell Viability Assay

The cells (1×10^5^ cells/well) were cultured with or without the indicated stimulations for 72 hours in a U-bottom 96-well microtiter plate. Cell viability was then determined by trypan blue dye exclusion and a WST assay using the Tetra Color ONE kit (Seikagaku Corporation, Tokyo, Japan) according to the manufacturer’s instructions. To detect apoptotic cells, the spleen cells or MLN cells (1×10^6^) isolated from the mice were incubated with 5 µl FITC-conjugated Annexin V and 5 µl PI (BD Pharmingen, San Diego, CA, USA) in 500 µl PBS for 15 minutes. After washing with PBS, the cells were subjected to FACS analysis. For some experiments, the cells isolated from the mice were re-stimulated with 300 µg/ml OVA *in vitro* for 48 hours and stained with PI and Annexin V and subjected to FACS analysis.

### FACS

PE-conjugated anti-mouse KJ1–26, FITC-conjugated anti-mouse CD80 and CD86, and APC-conjugated anti-mouse CD11c antibody were purchased from BD PharMingen (San Diego, CA). APC-conjugated anti-mouse CD25 antibody were purchased from eBioscience Inc. (San Diego, CA). The cellular staining was analyzed on a FACSCalibur (BD Biosciences, San Diego, CA) using the CellQuest program (BD Biosciences). Before staining, Fc receptors were blocked with anti-CD16/32 antibody (2.4G2; BD Biosciences). Negative controls consisted of isotype-matched directly conjugated, nonspecific antibodies (BD Biosciences).

### cAMP Assay

cAMP was quantified using a cAMP EIA kit (Cayman Chemical Company, Ann Arbor, MI). Resveratrol (10 and 30 µM) was added to BMDCs (5 × 10^5^ cells/well) grown in 24-well plates and incubated at 37°C for 30 minutes before addition of 12 pM CT or 10 nM prostaglandin E2 (PGE2) (Tokyo Chemical Industry Co., Ltd., Tokyo, Japan) as a positive control and further incubation for 1 hour. The medium was then aspirated and 0.3 ml of 2% triton (Sigma-Aldrich, St Louis, MO) in EIA buffer (company supplied) was added. The cells were incubated for 10 min at room temperature and sedimented by centrifugation and the supernatant was used for assay following the manufacturer’s instructions.

### Oral Administration of a Lethal Dose of CT to Mice

Mice were deprived of food for 2 hours and were deprived of water for another 2 hours and then were given a solution of sodium bicarbonate to neutralize stomach acidity before oral administration of CT as previously described [19]. Then 30 minutes later, mice were mildly sedated by anesthesia and given oral challenge with 150 µg CT in 10 mM Tris, pH7.5; they were monitored daily after the challenge if they were allowed to die after the lethal dose.

### Data Analysis

The statistical analysis was performed using un-paired Student’s *t-*test. A value of P<0.05 was considered to be significant.

## Supporting Information

Figure S1 **The frequencies of apoptotic cells in splenocytes and mesenteric lymph node cells isolated from standard diet- or standard diet plus resveratrol-fed mice.** The splenocytes and mesenteric lymph node (MLN) cells were freshly isolated from the mice fed standard diet or fed the standard diet plus resveratrol at 5 weeks (day 35). A. B. The cells were immediately stained with propidium iodide (PI) and Annexin V and subjected to FACS analysis. Representative plots (left panels) and the quantitative data (right panels) of the splenocytes (SPL) (A) and MLN cells (B). C. The cells were stimulated with 300 µg/ml OVA *in vitro* for 2 days and were stained with PI and Annexin V and subjected to FACS analysis. The quantitative data of the splenocytes (SPL) and MLN cells are shown. Values represent the mean ± SD (A, B, n = 4 per group, C, n = 3 per group). (S: standard diet-fed mice, S/R: standard diet plus resveratrol-fed mice).
(TIF)

Figure S2 **The frequencies of viable cells in DO11.10 splenocytes stimulated in the presence or absence of resveratrol.** The splenocytes isolated from DO11.10 mice were stimulated with 300 µg/ml OVA +12 pM CT in the presence or absence of 10 or 30 µM resveratrol for 72 hours. The cells were then stained with trypan blue solution. Trypan blue exclusion rates relative to those in control group (OVA + CT stimulation) are shown. Values represent the mean ± SD (n = 4 per group).
(TIF)
