# Supplementary figures and images for: Dietary Resveratrol Prevents the Development of Food Allergy in Mice
Source: PLoS One. 2012 Sep 4;7(9):e44338. doi: 10.1371/journal.pone.0044338 (PMC3433457; doi:10.1371/journal.pone.0044338)

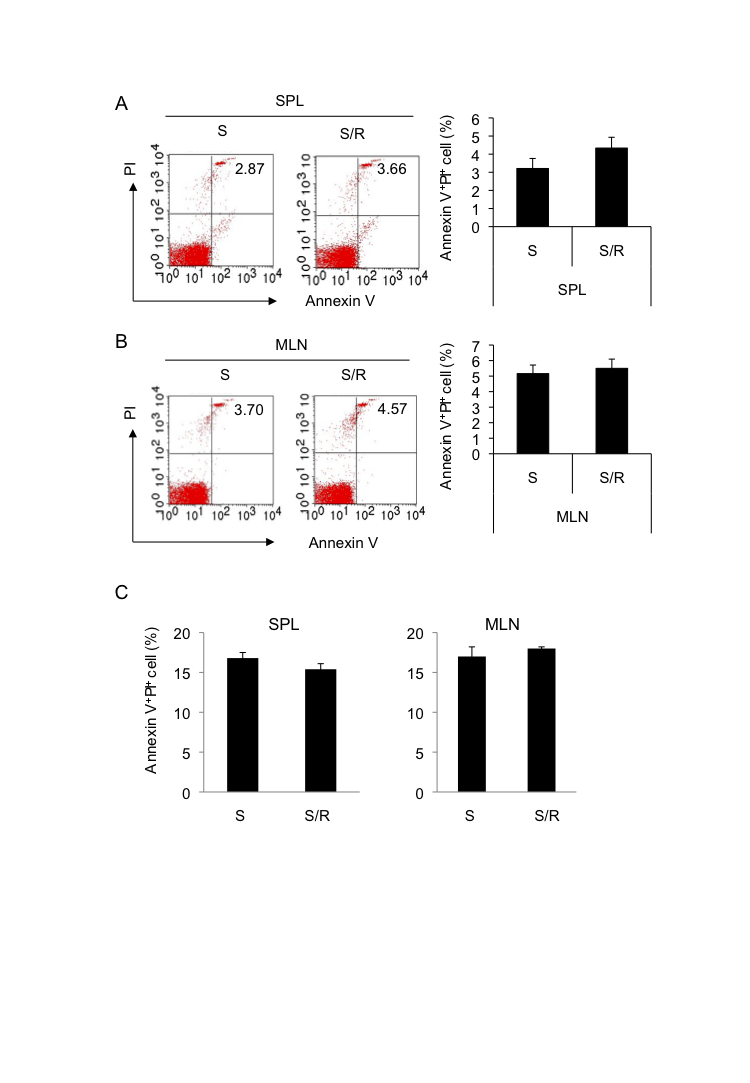

Supplement: Figure S1 — The frequencies of apoptotic cells in splenocytes and mesenteric lymph node cells isolated from standard diet- or standard diet plus resveratrol-fed mice. The splenocytes and mesenteric lymph node (MLN) cells were freshly isolated from the mice fed standard diet or fed the standard diet plus resveratrol at 5 weeks (day 35). A. B. The cells were immediately stained with propidium iodide (PI) and Annexin V and subjected to FACS analysis. Representative plots (left panels) and the quantitative data (right panels) of the splenocytes (SPL) (A) and MLN cells (B). C. The cells were stimulated with 300 µg/ml OVA in vitro for 2 days and were stained with PI and Annexin V and subjected to FACS analysis. The quantitative data of the splenocytes (SPL) and MLN cells are shown. Values represent the mean ± SD (A, B, n = 4 per group, C, n = 3 per group). (S: standard diet-fed mice, S/R: standard diet plus resveratrol-fed mice). (TIF) [file pone.0044338.s001.tif]

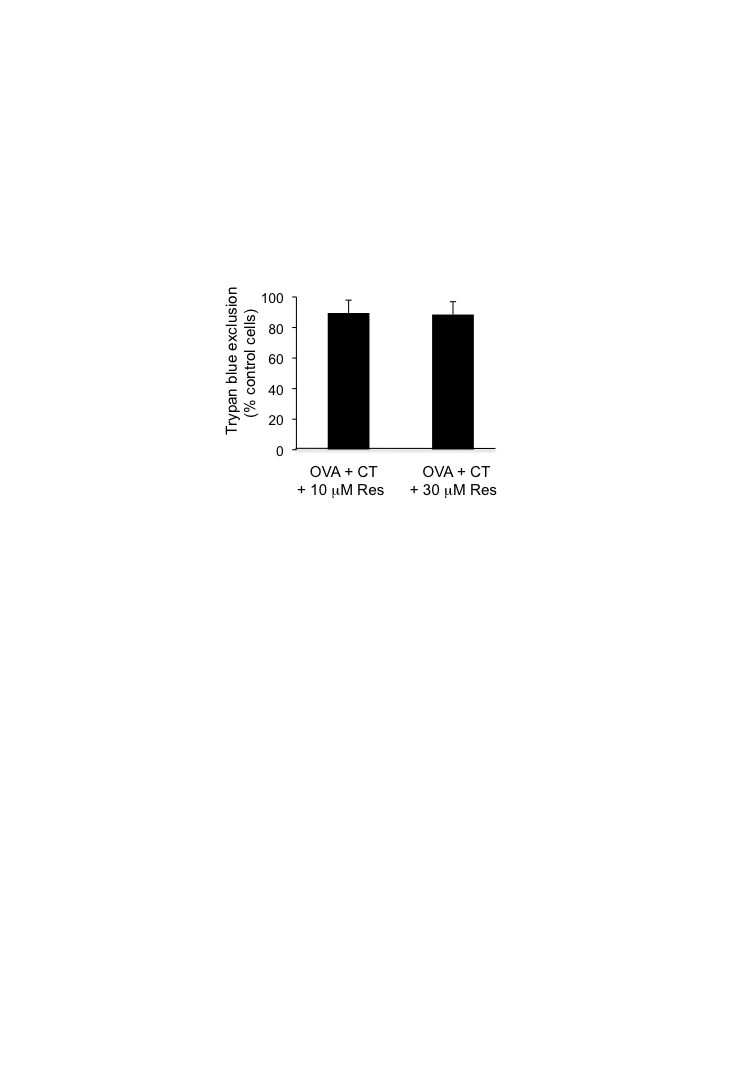

Supplement: Figure S2 — The frequencies of viable cells in DO11.10 splenocytes stimulated in the presence or absence of resveratrol. The splenocytes isolated from DO11.10 mice were stimulated with 300 µg/ml OVA +12 pM CT in the presence or absence of 10 or 30 µM resveratrol for 72 hours. The cells were then stained with trypan blue solution. Trypan blue exclusion rates relative to those in control group (OVA + CT stimulation) are shown. Values represent the mean ± SD (n = 4 per group). (TIF) [file pone.0044338.s002.tif]
